# Next generation phenotyping for diagnosis and phenotype–genotype correlations in Kabuki syndrome

**DOI:** 10.1038/s41598-024-52691-3

**Published:** 2024-01-28

**Authors:** Quentin Hennocq, Marjolaine Willems, Jeanne Amiel, Stéphanie Arpin, Tania Attie-Bitach, Thomas Bongibault, Thomas Bouygues, Valérie Cormier-Daire, Pierre Corre, Klaus Dieterich, Maxime Douillet, Jean Feydy, Eva Galliani, Fabienne Giuliano, Stanislas Lyonnet, Arnaud Picard, Thantrira Porntaveetus, Marlène Rio, Flavien Rouxel, Vorasuk Shotelersuk, Annick Toutain, Kevin Yauy, David Geneviève, Roman H. Khonsari, Nicolas Garcelon

**Affiliations:** 1 Imagine Institute, INSERM UMR1163, 75015 Paris, France; 2 Service de chirurgie maxillo-faciale et chirurgie plastique, Hôpital Necker-Enfants Malades, Assistance Publique-Hôpitaux de Paris, Paris, France; 3 Centre de Référence des Malformations Rares de la Face et de la Cavité Buccale MAFACE, Filière Maladies Rares TeteCou, Paris, France; 4 Faculté de Médecine, Université de Paris Cité, 75015 Paris, France; 5 Laboratoire ‘Forme et Croissance du Crâne’, Faculté de Médecine, Hôpital Necker-Enfants Malades, Assistance Publique-Hôpitaux de Paris, Université Paris Cité, Paris, France; 6 Hôpital Necker-Enfants Malades, 149 rue de Sèvres, 75015 Paris, France; 7 Département de Génétique Médicale, Maladies Rares et Médecine Personnalisée, Génétique clinique, CHU Montpellier, Centre de référence anomalies du développement SOOR, INSERM U1183, Montpellier University, Montpellier, France; 8 Service de médecine génomique des maladies rares, Hôpital Necker-Enfants Malades, Assistance Publique-Hôpitaux de Paris, Paris, France; 9 Service de Génétique, CHU Tours, UMR 1253, iBrain, Université de Tours, Inserm, Tours, France; 10 Nantes Université, CHU Nantes, Service de chirurgie maxillo-faciale et stomatologie, 44000 Nantes, France; 11 Nantes Université, Oniris, UnivAngers, CHU Nantes, INSERM, Regenerative Medicine and Skeleton, RMeS, UMR 1229, 44000 Nantes, France; 12 Univ. Grenoble Alpes, Inserm, U1209, IAB, CHU Grenoble Alpes, 38000 Grenoble, France; 13 HeKA team, INRIA, 75012 Paris, France; 14 MEDISYN Genetics, Lausanne, Switzerland; 15 Center of Excellence in Genomics and Precision Dentistry, Department of Physiology, Faculty of Dentistry, Chulalongkorn University, Bangkok, Thailand; 16 Center of Excellence for Medical Genomics, Department of Pediatrics, Faculty of Medicine, Chulalongkorn University, Bangkok, Thailand

**Keywords:** Medical genetics, Genetic counselling, Computer science

## Abstract

The field of dysmorphology has been changed by the use Artificial Intelligence (AI) and the development of Next Generation Phenotyping (NGP). The aim of this study was to propose a new NGP model for predicting KS (Kabuki Syndrome) on 2D facial photographs and distinguish KS1 (KS type 1, *KMT2D*-related) from KS2 (KS type 2, *KDM6A*-related). We included retrospectively and prospectively, from 1998 to 2023, all frontal and lateral pictures of patients with a molecular confirmation of KS. After automatic preprocessing, we extracted geometric and textural features. After incorporation of age, gender, and ethnicity, we used XGboost (eXtreme Gradient Boosting), a supervised machine learning classifier. The model was tested on an independent validation set. Finally, we compared the performances of our model with DeepGestalt (Face2Gene). The study included 1448 frontal and lateral facial photographs from 6 centers, corresponding to 634 patients (527 controls, 107 KS); 82 (78%) of KS patients had a variation in the *KMT2D* gene (KS1) and 23 (22%) in the *KDM6A* gene (KS2). We were able to distinguish KS from controls in the independent validation group with an accuracy of 95.8% (78.9–99.9%, *p* < 0.001) and distinguish KS1 from KS2 with an empirical Area Under the Curve (AUC) of 0.805 (0.729–0.880, p < 0.001). We report an automatic detection model for KS with high performances (AUC 0.993 and accuracy 95.8%). We were able to distinguish patients with KS1 from KS2, with an AUC of 0.805. These results outperform the current commercial AI-based solutions and expert clinicians.

## Introduction

Kabuki syndrome (KS) is a rare genetic disorder, with an estimated prevalence of 1:86,000 to 1:32,000^1–3^. The typical KS face includes long palpebral fissures associated with eversion of the lateral third of the lower eyelid; long and heavy lashes giving the impression of made-up eyes; broad, arched and interrupted eyebrows; broad, depressed nasal tip; and prominent, cupped ears^1,2,4^. Extra-facial features include mild to moderate intellectual disability, visceral malformations, skeletal dysplasia and immunological manifestations^5^. KS has been described in all ethnic groups^6,7^.

More than 80% of KS patients have a pathogenic variant in the coding regions of *KMT2D* (KS type 1, KS1, OMIM147920), and around 10% of patients have a pathogenic variant in the *KDM6A* gene (KS type 2, KS2, OMIM300128)^8–12^.

Improving syndrome screening in clinical genetics is a crucial challenge in reducing diagnostic wandering. In France, the 7000 rare diseases identified to date represent 4.5% of the population, half of which affect children under the age of 5 with 10% of deaths between 0 and 5. Around 50% of patients are not diagnosed, and for the remaining 50%, diagnostic wandering reaches an average of 5 years^13^. Diagnostic wandering is defined by the failure to define the precise cause of a disease after having performed all available investigations. Applications of Artificial Intelligence (AI) are increasing in healthcare^14–17^. The field of dysmorphology has been changed by these new methods, under the name of Next Generation Phenotyping (NGP)^18^. Publications comparing human performances to NGP are flourishing^19–22^, and some suggest that digital tools do it better than human experts in terms of diagnosis: Dudding-Byth et al.^23^ showed a better performance of NGP compared to clinicians in a group of ten genetic syndromes, not including KS; Rouxel et al.^5^ compared the performance of the DeepGestalt technology^18^ using the Face2Gene online tool (FDNA Inc. Boston, MA, USA) to the performances of clinicians trained in the recognition of KS1 and KS2.

The aim of this study was to develop a NGP model for the diagnosis of KS and for distinguishing KS1 from KS2. We trained and validated the model on a large national and international multi-center cohort of patients of all ages and ethnicities. The specificity of this approach was the integration of lateral pictures, including the outline of the cranial vault and the position of the ears, as well as frontal pictures and the morphology of the external ear.

## Materials and methods

The study was approved by the Comité Éthique et Scientifique pour les Recherches, les Études et les Évaluations dans le domaine de la Santé (CESREES), №4570023bis, the Commission Nationale Informatique et Libertés (CNIL), №MLD/MFI/AR221900, the Institutional Review Board, Faculty of Medicine, Chulalongkorn University (IRB 264/62), and in accordance with the 1964 Helsinki declaration and its later amendments. Informed and written consents were obtained from the legal representatives of each child or from the patients themselves if they were of age.

### Photographic dataset

We included most pictures from the photographic database of the Maxillofacial surgery and Plastic surgery department of *Hôpital Necker—Enfants Malades* (Assistance Publique—Hôpitaux de Paris), Paris, France. This database contains 594,000 photographs from 22,000 patients, and all pictures since 1995 were taken by a professional medical photographer using a Nikon D7000 device in standardized positions.

We included retrospectively and prospectively, from 1995 to 2023, all frontal and lateral pictures of patients diagnosed with KS. The photographs were not calibrated. All patients had genetic confirmation of KS (*KMT2D* or *KDM6A*). We excluded all photographs taken after any surgerical procedure that could have modified the craniofacial morphology. Multiple photographs per patient corresponded to different ages of follow-up. Duplicates were excluded.

Controls were selected among patients admitted for lacerations, trauma, infection and various skin lesions, without any record of chronic conditions. More precisely, follow-up for any type of chronic disease was considered as an exclusion criterion. The reports were retrieved using the local data warehouse Dr Warehouse^24^. For each patient, the best lateral view was included.

Data from five other medical genetics departments were also included according to the same criteria: (1) Montpellier University Hospital (n = 32), (2) Grenoble University Hospital (n = 1), (3) Tours University Hospital (n = 1), (4) King Chulalongkorn Memorial Hospital Bangkok, Thailand (n = 8), and (5) Lausanne University Hospital, Lausanne, Switzerland (n = 1).

### Validation set

For designs №1 and №2, we randomly selected a group of individuals corresponding to 10% of the number of patients with KS, and the equivalent number of control patients. These patients were removed from the training set. The two sets were therefore independent.

### Landmarking

We used three different templates based on 105 landmarks for the frontal views, 73 for the lateral views and 41 for the external ear pictures. We developed an automatic annotation model for each template following a pipeline including: (1) detection of the Region Of Interest (ROI) and (2) automatic placement of the landmarks.

For ROI detection, a Faster Region-based Convolutional Neural Network (RCNN) model was trained after data augmentation (images and their + 10° and  10° rotations), with a learning rate of 0.001, a batch size of 4, a gamma of 0.05 and 2000 iterations, optimized and split into two stages: ROI detection and determination of profile laterality.

(1) *ROI detection—*Faster RNN trained on 15,633 images, after data augmentation (images and their + 10° and − 10° rotations): 6186 frontal images (2062 × 3) and 9447 right and left profile images (3159 × 3). The batch size was 2, learning rate was 0.0025, and the maximum number of iterations was 2800.

(2) *Determination of profile laterality*—Pre-trained ResNet50 network^25^ using the Pytorch library^26^. The training images included 1570 left profiles and 1579 right profiles. The batch size was 16, an Adam optimizer^27^ was used with a learning rate of 0.001, a step of 7, and a gamma of 0.1, trained over 25 epochs.

For the automatic placement of landmarks, we used a patch-based Active Appearance Model (AAM) using the *menpo* library on Python 3.7^28^. We have previously reported the relevance of this approach^29^. We used two-scale landmarking*:* the model for frontal pictures was trained on 904 manually annotated photographs, with a first stage of dimensioning (diagonal = 150), a patch shape of [(15, 15), (23, 23)] and 50 iterations and a second stage without resizing, with a patch shape of [(20, 20), (30, 30)] and 10 new iterations. The model for profile pictures was trained on 1,439 manually annotated photographs, with a first stage of dimensioning (diagonal = 150), a patch shape of [(15, 15), (23, 23)] and 25 iterations and a second stage without resizing, with a patch shape of [(15, 15), (23, 23)] and 5 new iterations. The model for ears was trained on 1221 manually annotated photographs, with a first stage of dimensioning (diagonal = 100), a patch shape of [(15, 15), (23, 23)] and 50 iterations and a second stage without resizing, with a patch shape of [(20, 20), (30, 30)] and 20 new iterations. All three models used the Lucas Kanade optimizer^30^.

Each automatically annotated photograph was checked by two authors blinded for the diagnosis, QH and MD, and landmarks were manually re-positioned when necessary, using *landmarker.io*^31^. The Intraclass Correlation Coefficient (ICC) was computed between the raters. ICC values greater than 0.9 corresponded to excellent reliability of the manual annotation^32^.

### Geometric morphometrics

We performed Generalized Procrustes Analysis (GPA)^33^ on all landmark clouds using the *geomorph* package on R^34^. Since the data were uncalibrated photographs, ROI sizes were not available: shape parameters only were assessed and not centroid sizes. Procrustes coordinates were processed using Principal Component Analysis (PCA) for dimension reduction. We retained the principal components explaining 99% of the total variance in cumulative sum. The last 1% was considered as negligible information.

### Texture extraction

We partitioned the frontal and profile pictures into key areas and applied textural feature extraction methods to each zone, allowing to check the results and determine which zone had contributed most to the diagnosis.

We defined 14 key areas that could potentially contribute to diagnosis: 11 on frontal views (right/left eyes, right/left eyebrows, glabella, forehead, nasal tip, philtrum, right/left cheeks, and chin) and 3 on lateral views (pre-auricular region, eye, and zygoma relief). Each zone was extracted automatically using the previously placed landmarks.

We used the Contrast Limited Adaptative Histogram Equalization (CLAHE) algorithm for histogram equalization, as previously reported before the use of feature extractors^35,36^. CLAHE enhanced contrast by evenly dispersing gray values^37^ and by reducing the influences of illumination during picture capture and of skin color. Kiflie et al. recommended CLAHE as a first choice equalization method^38^.

Gray-Level Co-occurrence Matrix (GLCM) methods, as proposed by Haralick^39^, are based on the estimation of the second-order joint conditional probability density functions, which characterize the spatial relationships between pixels. GLCM is commonly used in texture analysis^40,41^, for instance in radiomics on CT-scan or MRI images^42–44^ or for skin texture assessment^45^. In GLCM, the co-occurrence matrix contains information on entropy, homogeneity, contrast, energy and correlation between pixels. GLCM includes 28 features, taking into account the average and range for each item of information and for each zone, representing 28 × 14 = 394 textural features for each patient.

### Stratification using metadata

The textural features and the geometric principal components were combined for further analysis. To consider associated metadata (age and gender) and the fact that we included more than one photograph per patient (that is the non-independence of the data), a mixed model was designed for each feature. The variables to be explained were the features (geometric and textural), with age, gender and ethnicity considered as explanatory variables. A random effect on age and individuals was introduced. The equation of the mixed model was:

$${\varvec{Features}}_{{{\varvec{i}},{\varvec{j}}}} \sim \alpha + age. \beta_{1} + gender.\beta_{2} + ethnicity.\beta_{3} + age.\beta_{1,i} + \varepsilon_{i,j}$$

where $age.\beta_{1,i}$ corresponded to a random slope for age per individual, and $\varepsilon_{i,j}$ was a random error term. We did not use an interaction term between age and gender and age and ethnicity as it did not increase the likelihood of the model. Age, gender and ethnicity are significant factors in dysmorphology^46,47^.

The residuals of each feature were computed to consider potential biases linked to the metadata:

$${\varvec{\varepsilon}}_{{{\varvec{i}},{\varvec{j}}}} = {\varvec{Features}}_{{{\varvec{i}},\user2{ j}}} - \alpha + age. \beta_{1} + gender.\beta_{2} + ethnicity.\beta_{3} + age.\beta_{1,i}$$

### Classification model

The inputs to the model were the residuals from the linear models described above, for each geometric or textural feature. We used eXtreme Gradient Boosting (XGBoost), a supervised machine learning classifier, for all the analyses^48^. We chose a tree-based booster, and the loss function to be minimized was a logistic regression for binary classification. We set several hyperparameters to improve the performance and effect of the machine learning model: learning rate = 0.3, gamma = 0, maximum tree depth = 6. The model with the lowest error rate was chosen for analysis. We separated the dataset into a training set and a testing set, and a five-fold cross-validation was used to define the ideal number of iterations to avoid overfitting.

The chosen model with the ideal number of iterations was then used on the independent validation set to test performances, by plotting accuracy and AUC. The Receiver Operating Characteristics (ROC) curves were plotted in R using the *plotROC* package^49^. We used the DeepGestalt tool proposed by Face2Gene CLINIC on our validation set, to be able to compare its performance (accuracies).

### Uniform Manifold Approximation and Projection (UMAP) representations

**Figure 1:**
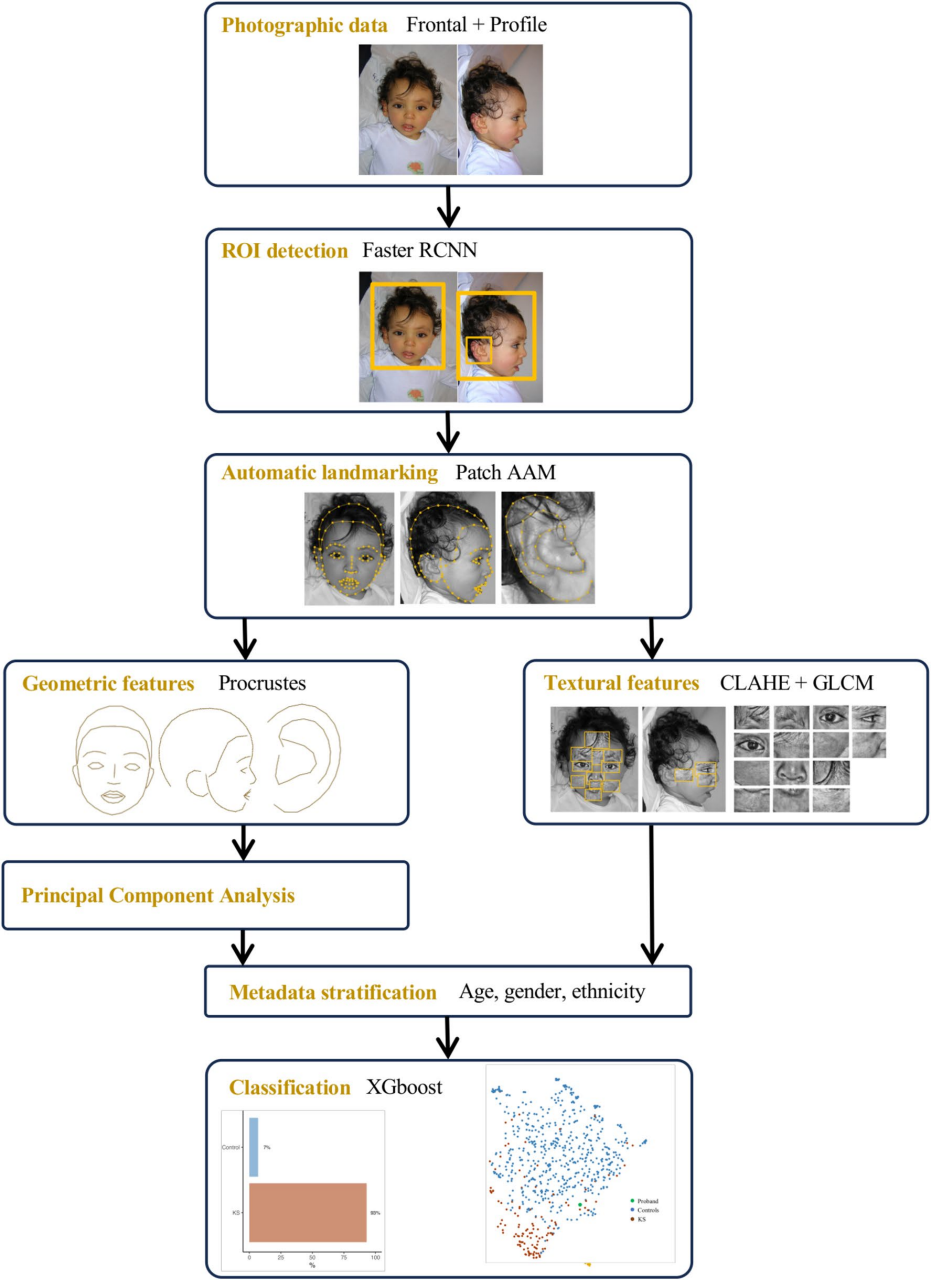
Analysis pipeline, from the initial photograph to diagnostic probability. ROI, Region Of Interest; AAM, active appearance model; Faster RCNN, Faster Region-based Convolutional Neural Network; CLAHE, Contrast Limited Adaptative Histogram Equalization; GLCM, Gray-Level Co-occurrence Matrix; XGboost, eXtreme Gradient Boosting.

The residuals $\varepsilon_{i,j}$ were represented using UMAP for visual clustering, a nonlinear dimension reduction technique^50^. We retained the residuals associated with features with a classification gain (in their cumulative sum) > 0.75 in the importance matrix associated with the XGboost model. A k (local neighborhood size) value of 15 was used. A cosine metric was introduced to compute distances in high dimensional spaces: the effective minimal distance between embedded points was $10^{ - 6}$. The three conditions of UMAP, namely uniform distribution, local constancy of the Riemannian metric and local connectivity were verified. UMAP analyses were performed using the package *umap* on R^51^ (Fig. 1).

### Classification designs

1. *Design №1*, syndrome diagnosis support: KS was tested against controls in a binary classification.
2. *Design №2*, genotype–phenotype correlations: KS1 and KS2 were tested in binary classifications.
3. *Design №3*, genotype–phenotype correlations: KS1 Protein-Altering Variants (PAVs) and Protein-Truncating Variants (PTVs) were tested in binary classifications.

### Ethics approval

This study was performed in line with the principles of the Declaration of Helsinki. Approval was granted by the CESREES (17/06/2021, 4570023).

### Consent to participate

Written informed consent was obtained from the parents.

### Consent to publish

The authors affirm that human research participants provided informed consent for publication of the images in Figs. 1, 4 and 7.

## Results

### Population description

Ranging between 1998 and 2023, we included 1448 frontal and lateral facial photographs, corresponding to 634 patients. The mean age was 7.2 ± 4.2 years and ranged from 0 to 40.2 years; 52% were girls. Ethnicity was 92% Caucasian, 6% African or Caribbean, and 3% Asian.

The control group comprised 1084 photographs, corresponding to 527 patients with a mean age of 7.0 ± 4.6 years. Fifty-four percent were girls and ethnicities were 93% Caucasian, 5% African/Caribbean, and 2% Asian.

The KS group comprised 364 photographs, corresponding to 107 patients with a mean age of 7.8 ± 6.7 years. Forty-two percent were girls and ethnicities were 85% Caucasian, 7% African/Caribbean, and 8% Asian. Seventy-eight percent of patients were KS1 (Table 1).

**Table 1:** Clinical description of the cohort.

		Total	Controls	KS
N				
	Consultations	724	542 (75%)	182 (25%)
	Photographs	1448	1084 (75%)	364 (35%)
	Patients	634	527 (83%)	107 (17%)
Gender				
	Female	331 (52%)	286 (54%)	45 (42%)
	Male	303 (48%)	241 (46%)	62 (58%)
Age (years)				
	Mean ± SD	7.2 ± 4.2	7.0 ± 4.6	7.8 ± 6.7
	Median	6.8	7.1	6.0
	Minimum	0	0.1	0
	Maximum	40.2	22.1	40.2
Ethnicity				
	African/Caribbean	35 (6%)	27 (5%)	8 (7%)
	Asian	18 (3%)	10 (2%)	8 (7%)
	Caucasian	581 (92%)	490 (93%)	91 (85%)
Genetic variation				
	*KMT2D*** (KS1)**			82 (78%)
	*KDM6A* **(KS2)**			23 (22%)

SD, standard deviation; KS, Kabuki Syndrome; *KMT2D*, Lysine (K)-specific methyltransferase 2D; *KDM6A*, Lysine (K)-specific demethylase 6A.

Two patients had a genetically confirmed diagnosis of KS, but we had no information on the causal gene. We thus collected information on genetic variation for 105 KS individuals with 82 (78%) and 23 (22%) with variations in *KMT2D* (KS1) and *KDM6A* (KS2) respectively.

In the KS1 group, 74% of variants were PTVs, with 49% nonsense variants leading to a premature stop codon (24% non-sense, 24% frameshift) and 26% splice donor site variants. Eighteen percent were PAVs, with 17% missense variants and 1% in-frame indel.

In the KS2 group, 78% of variants were PTVs, with 43% nonsense variants leading to a premature stop codon (30% non-sense, 13% frameshift), 30% splice donor site variants and 4% a large deletion. Nine percent were missense PAVs (Table 2).

**Table 2:** Molecular description of the cohort.

				Amino-acids	Nucleotides	Exon	N (%)
*KMT2D*							82 (78%)
	PTV						61 (74%)
		Nonsense					40 (49%)
			Nonsense				20 (24%)
				p.Cys247*	c.741T>A	7	1
				p.Ser286*	c.855_856del	7	1
				p.Gln1773*	c.5317C>T	22	1
				p.Gln1949*	c.5845C>T	27	1
				p.Gln2109*	c.6325C>T	31	1
				p.Arg2645*	c.7933C>T	31	1
				p.Arg2801*	c.8401C>T	34	1
				p.Gln3910*	c.11728C>T	39	1
				p.Gln3942*	c.11824C>T	39	1
				p.Gln4223*	c.12667C>T	39	1
				p.Gln4230*	c.12688C>T	39	2
				p.Arg4484*	c.13450C>T	39	1
				p.Arg4904*	c.14710C>T	48	1
				p.Arg5282*	c.15844C>T	49	1
				p.Tyr5321*	c.15963T>G	50	1
			Frameshift				20 (24%)
				p.Leu656Profs*12	c.1966dup	10	1
				p.Glu1224Argfs*26	c.3669dup	11	1
				p.Gly1235Valfs*95	c.3699del	11	1
				p.Met1379Valfs*52	c.4135_4136del	14	1
				p.Asp1876Glyfs*38	c.5627_5630del	25	1
				p.Ser2039Glnfs*8	c.6115del	29	2
				p.Ala2119Argfs*36	c.6354dup	31	1
				p.Pro2330Serfs*47	c.6987_6988insT	31	1
				p.Phe3672Leufs*76	c.11016_11019del	39	1
				p.Met3894Trpfs*85	c.11679del	39	1
				p.Glu4039Glyfs*17	c.12116_12117del	39	1
				p.Ser4138Cysfs*29	c.12413_12414del	39	1
				p.Leu4483Cysfs*36	c.13446del	39	1
				p.Tyr5113Leufs*25	c.15337dup	48	1
				p.Lys5139Gly	c.15415_15418del	48	1
				p.Leu5318Serfs*14	c.15953_15956del	50	1
		Splice donor site					21 (26%)
					c.674-1G>A		1
					c.1258+1G>A		1
					c.2797+1G>C		1
					c.13531-2A>C		1
					c.14516-1G>C		1
	PAV						15 (18%)
		Missense					14 (17%)
				p.Ala2182Thr		10	1
				p.Glu1391Lys	c.4171G>A	14	1
				p.Arg5048Cys	c.15142C>T	48	1
				p.Arg5048His	c.15143G>A	48	1
				p.Arg5154Gln	c.15461G>A	48	2
				p.Arg5179His	c.15536G>A	48	2
				p.Arg5214Cys	c.15640C>T	48	1
				p.Gly5295Ala	c.15884G>C	49	1
				p.Arg5340Gln	c.16019G>A	50	3
				p.Arg5432Trp	c.16294C>T	51	1
		Indel					1 (1%)
				p.Val275Ser	c.822_825delinsGTAGGCT	7	1
*KDM6A*							23 (22%)
	PTV						18 (78%)
		Nonsense					10 (43%)
			Nonsense				7 (30%)
				p.Tyr109*	c.327_333del	3	1
				p.Arg172*	c.514C>T	6	1
				p.Gln692*	c.2074C>T	17	1
				p.Gln1037*	c.3109C>T	20	1
				p.Trp1221*	c.3663G>A	25	1
				p.Arg1279*	c.3835C>T	26	2
			Frameshift				3 (13%)
				p.Gln607Alafs*25	c.1818_1819del	16	1
				p.Thr613Tyrfs*8	c.1846_1849del	16	1
				p.Ser1091Metfs*12	c.3270_3273del	17	1
		Splice donor site					7 (30%)
					c.564+1G>T		1
					c.619+6T>C		1
					c.620-2A>G		1
					c.875+1G>A		1
					c.2939-1G>T		1
					c.2988+1G>C		1
					c.3366-8_3366-4del		1
		Large deletion					1 (4%)
					exons 1 and 2		1
	PAV						2 (9%)
		Missense					2 (9%)
				p.Arg481His	c.1442G>A	14	1
				p.Arg1255Trp	c.3763C>T	26	1

*KMT2D* , Lysine (K)-specific methyltransferase 2D; *KDM6A*, Lysine (K)-specific demethylase 6A; PTV, protein-truncating variant; PAV, protein-altering variant.

### Design №1 : KS vs controls

1. Phenotype

**Figure 2:**
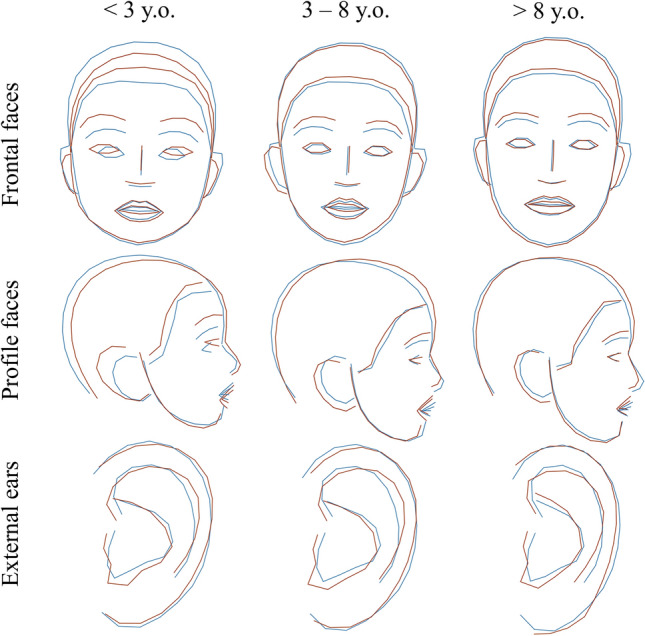
Average shapes in KS and controls and comparisons after Procrustes superimposition of frontal views, profile views, and external ears for three age groups. Blue = controls, Dark red = KS.

We confirmed the usual characteristics described in KS: high and arched eyebrows, long palpebral fissures, and large and prominent ears (Fig. 2).

2. Classification

**Figure 3:**
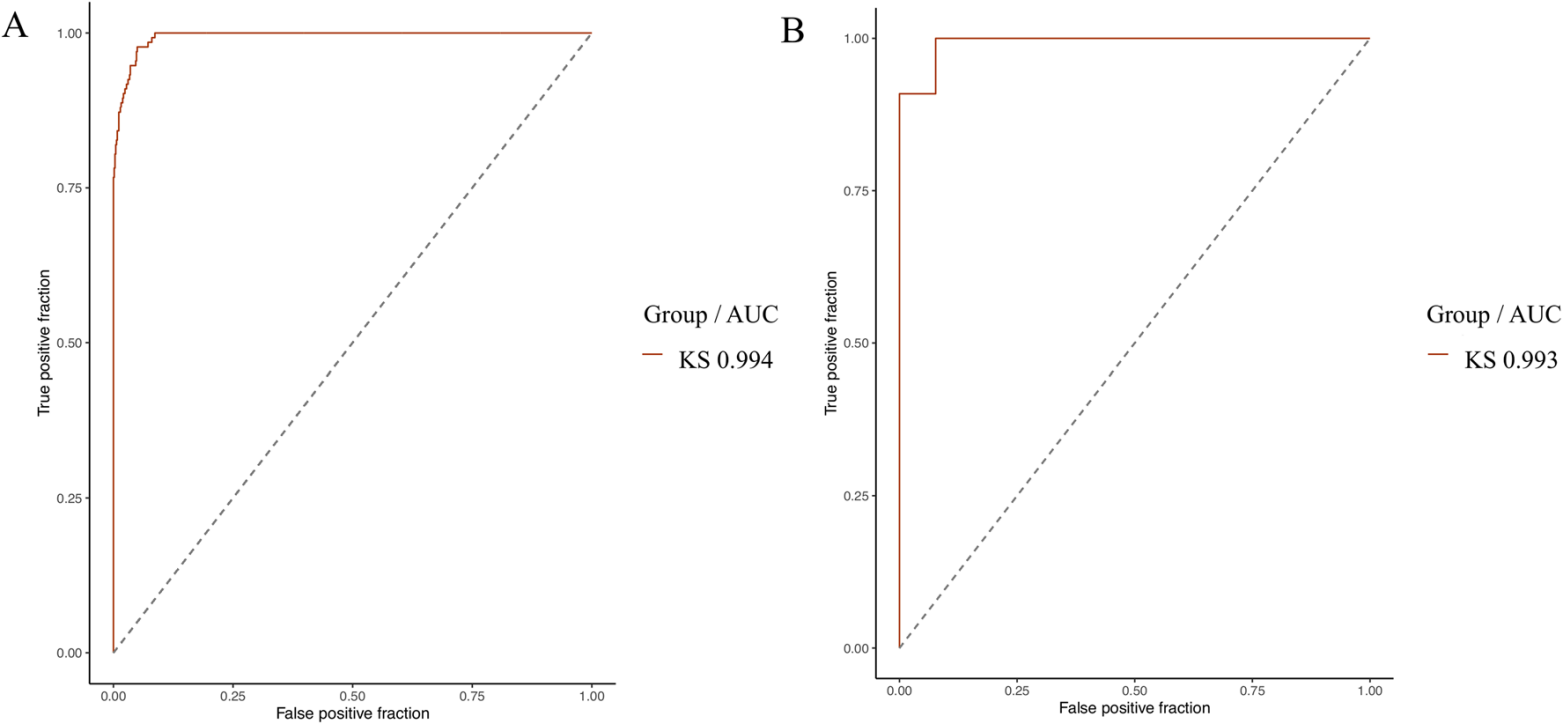
(**A**) Empirical ROC curves (training set) for KS with AUC in design №1. (**B**) ROC curves (validation set) for KS with AUC in design №1. AUC, area under the curve; KS, Kabuki Syndrome.

We were able to distinguish KS vs controls in the independent validation group with an accuracy of 95.8% (78.9–99.9%, *p* < 0.001). AUCs were comparable in the training set (0.994) and in the validation set (0.993) (Fig. 3, Table 3).

**Table 3:** Classification performances for design №1 (KS vs controls) in the validation group.

Design №1		
Accuracy	0.958 [0.789–0.999]	*p* < 0.001*
AUC	0.993 [0.974–1.000]	*p* < 0.001*
F1 score	0.963	

AUC,  area under the curve.

*Statistically significant (p < 0.05).

**Figure 4:**
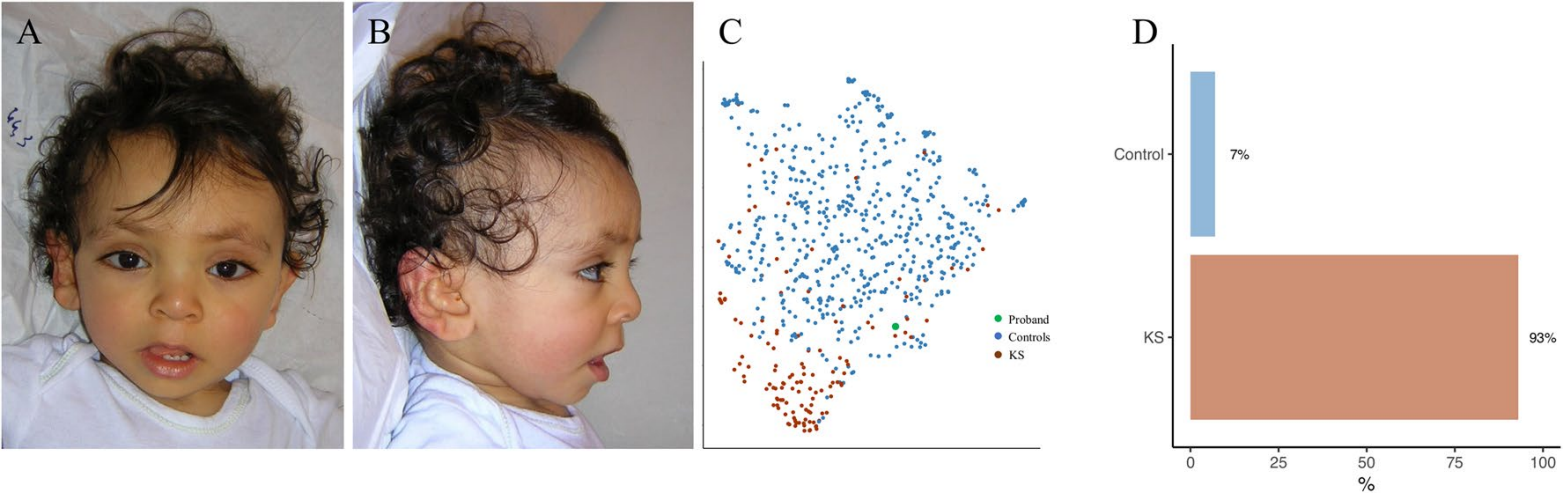
Classification using design №1 for proband 3 of the validation set. (**A**) and (**B**) Frontal and profile views of proband 3. (**C**) UMAP representation of the training data according to the two groups, with positioning of proband 3. (**D**) Histogram of predictions by the model. This child was also detected as KS by Face2Gene CLINIC. KS, Kabuki Syndrome.

Ten out of eleven patients were correctly predicted as KS with our model, and this performance was the same using Face2Gene CLINIC (Supp. Table 1). In addition, we were able to predict all control patients (Fig. 4, Table 4).

**Table 4:** Confusion matrix for design №1 (KS versus controls) in the validation group.

		Reference	
		Control	KS
Prediction	Control	**13**	1
	KS	0	**10**

Bold values: True Positives (TP).

KS, Kabuki Syndrome.

### Design №2 : KS1 vs KS2

1. Phenotype

**Figure 5:**
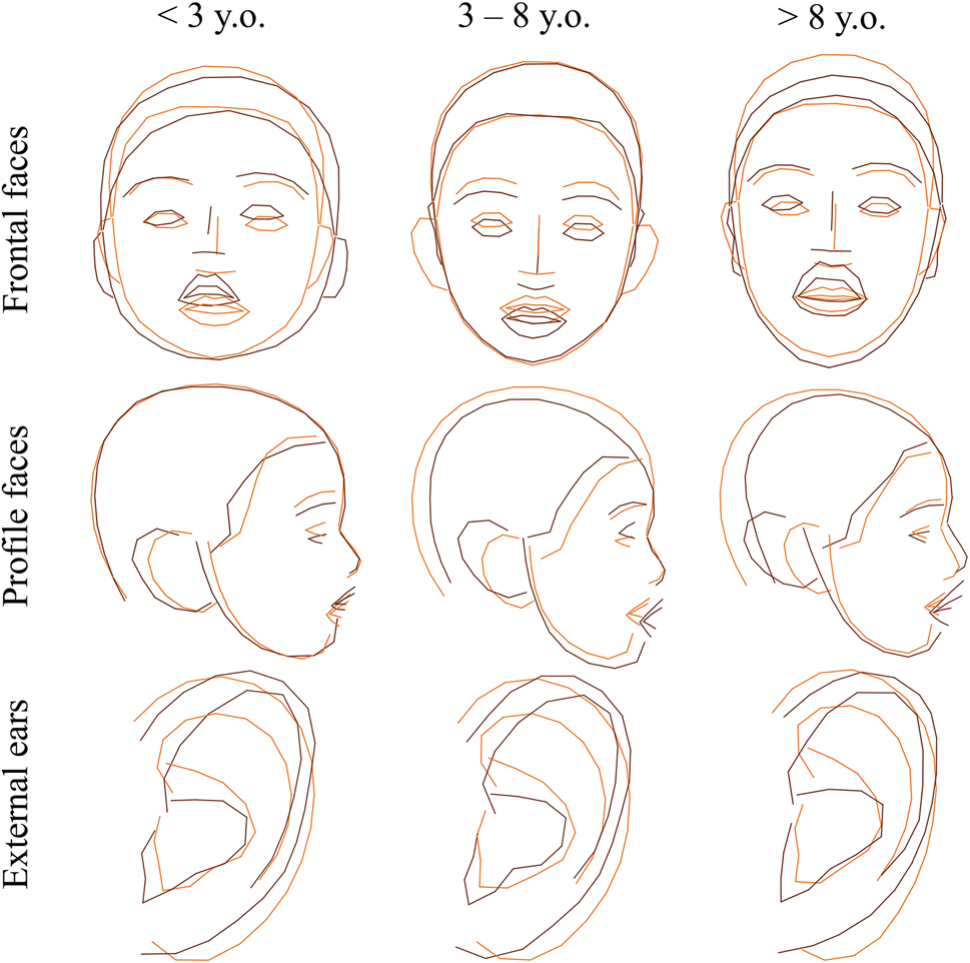
Average shapes in KS1 and KS2 and comparisons after Procrustes superimposition of frontal views, lateral views, and external ears for three age groups. Orange = KS1, Dark red = KS2.

KS2 individuals had a rounder face (HP:0000311), a shorter nose (HP:0003196), a thicker upper lip (HP:0000215), anteverted nostrils (HP:0000463), and a shorter midface (HP:0011800). There was no obvious difference in the eyebrows and eyes. The external ears were more elongated vertically in KS2 (HP:0400004), with a hypoplastic lobe (HP:0000385), and with a counter-clockwise rotation. The conch seemed more vertical in KS1 (Fig. 5).

2. Classification

**Figure 6:**
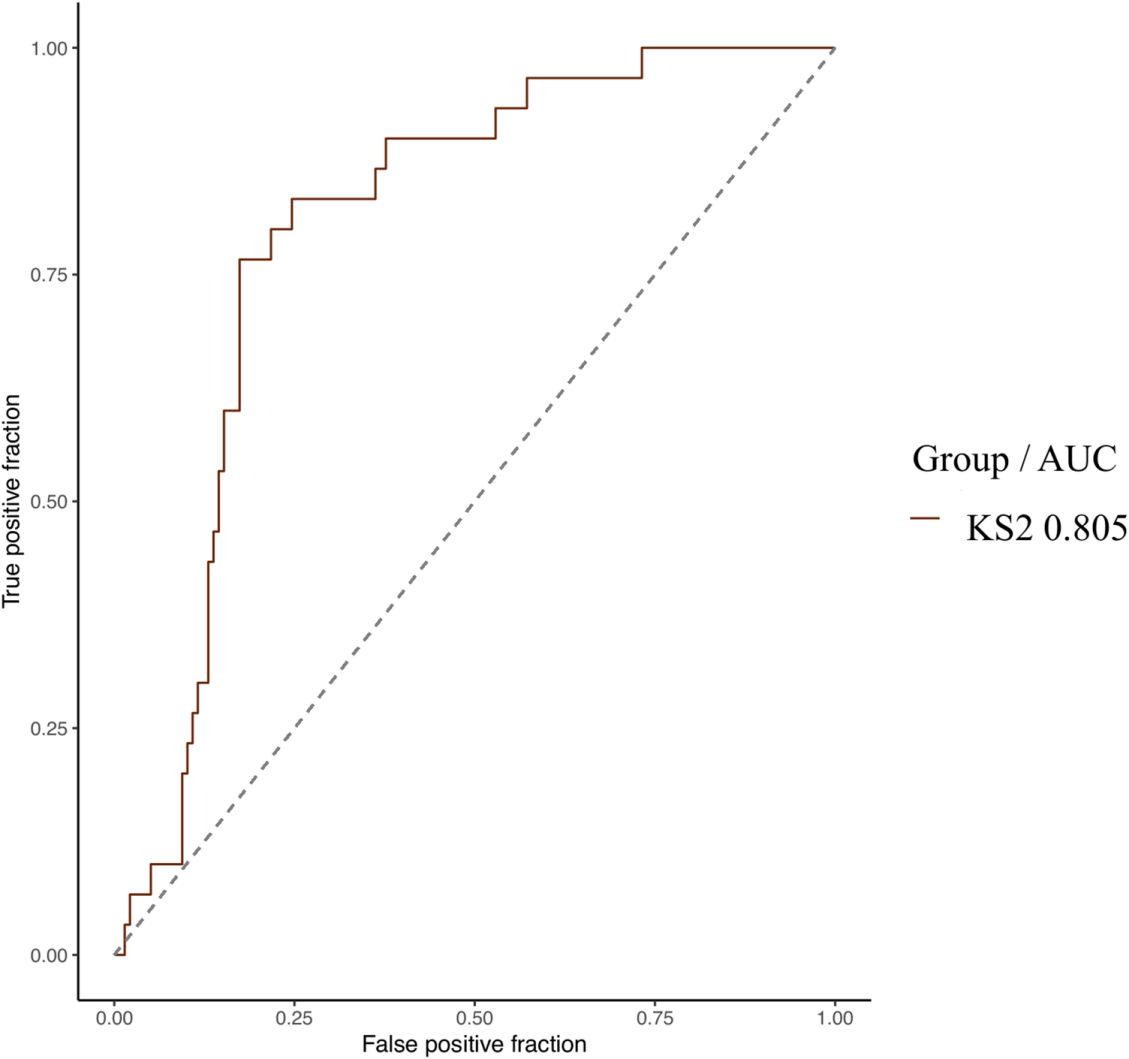
Empirical ROC curve (training set) for KS2 with AUC in design №2. AUC, Area Under the Curve; KS, Kabuki Syndrome.

**Figure 7:**
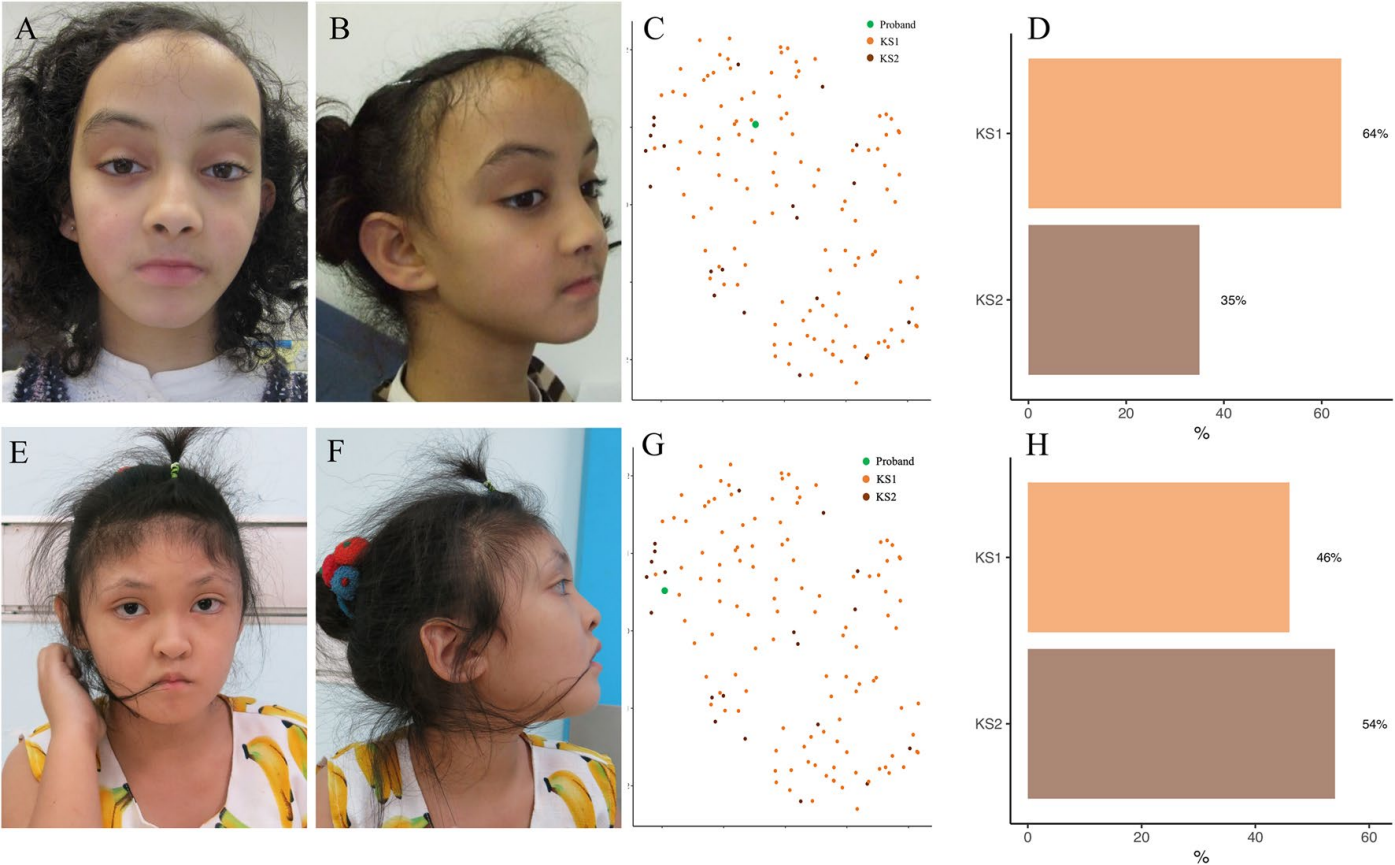
Classification using design №2 for two probands of the training set. (**A**, **B**, **E** and **F**) Frontal and profile views of the two probands. (**C** and **G**) UMAP representations of the training data according to the two groups, with positioning of probands 3. (**D** and **H**) Histograms of predictions by the model. The phenotype included a reduced height of the midface, a thicker upper lip, and a vertical elongation of the external ear in the KS2 group (**E** and **F**). KS, Kabuki Syndrome.

The model was able to distinguish KS1 from KS2 with an empirical AUC of 0.805 (0.729–0.880, p < 0.001) (Figs. 6, 7). This trend was found in the validation group, with an accuracy of 70% without reaching the significance threshold (Tables 5 and 6).

**Table 5:** Classification performances for design №2 (KS1 versus KS2) in the validation group.

Design №2		
Accuracy	0.700 [0.348–0.933]	*p* = 0.172
AUC	0.660 [0.314–1.000]	*p* = *0.221*
F1 score	0.727	

Significant values are in [italics].

AUC, area under the curve.

**Table 6:** Confusion matrix for design №2 (KS1 versus KS2) in the validation group.

		Reference	
		KS1	KS2
Prediction	KS1	**4**	2
	KS2	1	**3**

Bold values: True Positives (TP).

KS, Kabuki Syndrome.

### Design №3: PTV vs PAV in KS1

**Figure 8:**
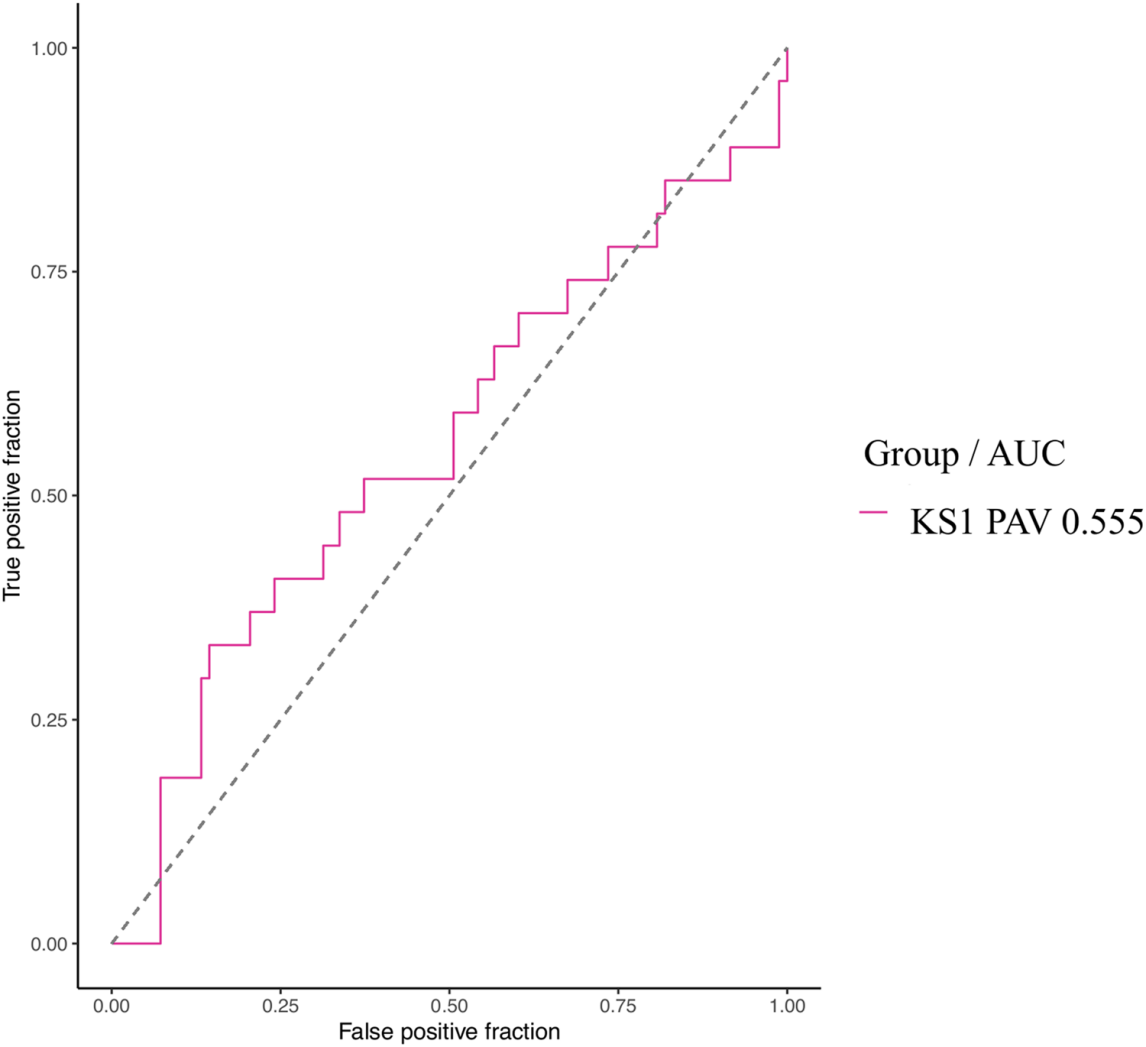
Empirical ROC curve (training set) for KS1 PAV with AUC in design №3. AUC, Area Under the Curve; KS, Kabuki Syndrome; PAV, protein-altering variant.

The model was unable to detect a difference in facial phenotype between KS1 patients with a PTV compared to KS1 patients with a PAV (0.555 [0.419–0.690], *p* = 0.786) (Fig. 8).

## Discussion

The model we report distinguished KS from controls in the independent validation group with an accuracy of 95.8% (78.9–99.9%, *p* < 0.001). Only 1 patient out of 24 was classified as ‘control’ while she had KS (accuracy 96%). In the KS group, 10 out of 11 patients were correctly classified (accuracy 91%). Using the Face2Gene CLINIC tool on KS patients (because DeepGestalt technology is not capable of recognizing non-syndromic patients) 1 patient out of 11 could not be analyzed and could not be classified as KS (accuracy 91%). Performances were therefore comparable. Interestingly, the patient not recognized by our model and by Face2Gene CLINIC was of African ethnicity, highlighting the lack of training data for non-Caucasian patients. The distribution of ethnic groups varies greatly from one center to another, which is why we believe it is important to encourage international collaborations in the field of Next Generation Phenotyping.

The model we report was also capable to distinguish KS1 from KS2 with an empirical AUC of 0.805 (0.729–0.880, p < 0.001). Rouxel et al.^5^ showed that the Face2Gene RESEARCH tool distinguished KS1 from KS2 in a cohort of 66 patients with an AUC of 0.722 (p = 0.022). The same team showed a classification accuracy of 61% (20/33) by clinical genetics experts between KS1 and KS2. The performance of our model was at least comparable to Face2Gene RESEARCH and seemed to outperform that of clinical experts.

Rouxel et al.^5^ explained that KS1 patients had a longer face and nose, a thin upper lip vermilion and a longer midface in comparison to KS2 patients, who have a rounder face, a thicker vermilion and anteverted nostrils. Our study reports new phenotypic features not seen on frontal images alone for KS2, such as a particular morphology of the external ear, longer along the vertical axis and with counter-clockwise rotation.

Phenotype-genotype correlations have been reported in KS for extra-facial anomalies. Cardiovascular abnormalities, namely ventricular septal defects, coarctation of the aorta, atrial septal defects, bicuspid aortic valve, patent ductus arteriosus, and hypoplastic left heart syndrome^52,53,53–55^ are more prevalent in KS2 compared to KS1^1,56^. Persistent hypoglycemia due to pituitary hormone deficiency, adrenal insufficiency, growth hormone deficiency and dysregulated insulin secretion by the pancreatic β-cells^57,58^ are also more frequent in KS2^10^, possibly because the inhibition of *KDM6A* increases the release of insulin from pancreatic islet cells, as suggested by mouse models^1,59^. Urinary tract anomalies, such as horseshoe kidneys and renal hypoplasia, seem to be more frequent in KS1, and genital defects such as cryptorchidism and hypospadias could be more frequent in KS2^56,60,61^.

Rouxel et al.^5^ underline the lack of Asian patients in their evaluation, and proposed that larger series were needed to better define phenotypical differences between KS1 and KS2, and the general dependance of the phenotype with ethnicity^6,12^. The collaboration with an Asian clinical genetics center (Bangkok) is thus a strong point of this study.

The use of textural feature extraction allowed our model account for typical KS characteristics not recognized by geometric analysis (Procrustes) alone. The lateral sparsening of the eyebrows and heavy lashes giving the impression of make-up eyes were thus included into in the classification.

Barry et al.^1^ reported a large meta-analysis including 152 articles and 1369 individuals with KS and assessed the prevalence of the different types of pathogenic variation per gene. The majority of *KMT2D* variants were truncating (non-sense 34%, frameshift 34%), then missense (23%) and finally splice site variants (9%). The majority of *KDM6A* variants were truncating (frameshift 36% > non-sense 27%), followed by splice site (20%), and missense (18%). We found similar results, with a higher prevalence of truncating non-sense variants for both genes. There was a higher prevalence of splice donor site variants, with 26% for *KMT2D* and 30% for *KDM6A*. Some authors report a more severe clinical outcomes in patients with non-sense variants than in patients with a frameshift variant^1^. Faundes et al.^56^ found more severe neurodevelopmental anomalies in patients with protein-truncating mutations in the KS2 group. Shah et al.^62^ reported ophthalmological anomalies such as strabismus, blue sclerae, microphthalmia and refractive anomalies that were more severe in patients with a non-sense variant, and less frequent in patients with a frameshift variant. Our model did not find any significant difference in facial phenotype between PTV and PAV.

## Conclusion

Here we report an automatic detection model for KS including the face, profiles and ears, with performances (AUC 0.993 and accuracy 95.8%) comparable to those of Face2Gene, on an independent validation set. These performances were achieved using an international cohort of 107 patients with a confirmed molecular diagnosis of KS. Using the same model, we were able to separate patients with KS1 (*KMT2D*) from KS2 (*KDM6A*), with an AUC of 0.805. These results seem to at least outperform Face2Gene and support the possibility of using a phenotype-first strategy to diagnose KS and detect its two causal genes.

### Supplementary Information

Supplementary Table 1.

## Data Availability

The code is available to readers on the website https://framagit.org/imagine-plateforme-bdd/mfdm/. The datasets (photographs) supporting the current study have not been deposited in a public repository because of their identifiable nature.
