# Supplementary material for: Next generation phenotyping for diagnosis and phenotype–genotype correlations in Kabuki syndrome
Source: Sci Rep. 2024 Jan 28;14:2330. doi: 10.1038/s41598-024-52691-3 (PMC10822856; doi:10.1038/s41598-024-52691-3)
Supplement: Supplementary file 1 — Supplementary Table 1. [file 41598_2024_52691_MOESM1_ESM.docx]

| **Proband** | **True group** | **Gender** | **Ethnicity** | **Subgroup** | **Our model** | **Face2Gene CLINIC** |
| --- | --- | --- | --- | --- | --- | --- |
| **1** | KS | F | Caucasian | KS1 | KS | KS |
| **2** | KS | F | Caucasian | KS1 | KS | KS |
| **3** | KS | M | Caucasian | KS1 | KS | KS |
| **4** | KS | F | Caucasian | KS2 | KS | KS |
| **5** | KS | M | Caucasian | KS1 | KS | KS |
| **6** | KS | M | Caucasian | KS2 | KS | KS |
| **7** | KS | M | Caucasian | KS1 | KS | KS |
| **8** | KS | F | African | KS1 | Control | Non analysable |
| **9** | KS | F | African | KS2 | KS | KS |
| **10** | KS | M | Caucasian | KS2 | KS | KS |
| **11** | KS | F | Caucasian | KS2 | KS | KS |

***Supplementary Table 1*. Comparison of predictions by the model with Face2Gene CLINIC for KS patients in the validation group.** KS = Kabuki Syndrome.
